# Effect of a Mediterranean Diet Intervention on Dietary Glycemic Load and Dietary Glycemic Index: The PREDIMED Study

**DOI:** 10.1155/2014/985373

**Published:** 2014-09-11

**Authors:** Ana Isabel Rodríguez-Rejón, Itandehui Castro-Quezada, Cristina Ruano-Rodríguez, María Dolores Ruiz-López, Almudena Sánchez-Villegas, Estefanía Toledo, Reyes Artacho, Ramón Estruch, Jordi Salas-Salvadó, María Isabel Covas, Dolores Corella, Enrique Gómez-Gracia, José Lapetra, Xavier Pintó, Fernando Arós, Miquel Fiol, Rosa María Lamuela-Raventós, Valentina Ruiz-Gutierrez, Helmut Schröder, Emilio Ros, Miguel Ángel Martínez-González, Lluis Serra-Majem

**Affiliations:** ^1^Research Institute of Biomedical and Health Sciences, University of Las Palmas de Gran Canaria, Luis Pasteur s/n, 35016 Las Palmas de Gran Canaria, Spain; ^2^Department of Nutrition and Food Science, School of Pharmacy, University of Granada, Campus Universitario de la Cartuja, 18071 Granada, Spain; ^3^Ciber Fisiopatología Obesidad y Nutrición (CIBEROBN, CB06/03), Instituto de Salud Carlos III, Edificio D 1*º* Planta, Hospital Clínico Universitario de Santiago de Compostela Choupana s/n, 15706 Santiago de Compostela, Spain; ^4^Department of Preventive Medicine and Public Health, School of Medicine, University of Navarra, Irunlarrea 1, Pamplona, 31080 Navarra, Spain; ^5^Department of Internal Medicine, Hospital Clinic, Institut d'Investigacions Biomèdiques August Pi Sunyer (IDIBAPS), Villarroel 170, 08036 Barcelona, Spain; ^6^Human Nutrition Department, School of Medicine, University Rovira i Virgili, Sant Llorenc 21, Reus, 43201 Tarragona, Spain; ^7^Cardiovascular Risk and Nutrition Research Group, Institut Municipal d'Investigació Medica (IMIM), Institut de Recerca del Hospital del Mar, Dr. Aiguader 88, 08003 Barcelona, Spain; ^8^Department of Preventive Medicine, School of Medicine, University of Valencia, Avenida Blasco Ibáñez 15, 46010 Valencia, Spain; ^9^Department of Preventive Medicine, School of Medicine, University of Malaga, Campus de Teatinos s/n, 29071 Malaga, Spain; ^10^Department of Family Medicine, Primary Care Division of Sevilla, San Pablo Health Center, Damasco s/n, 41007 Sevilla, Spain; ^11^Internal Medicine Service, Hospital of Bellvitge, c/Feixa Llarga s/n, L'Hospitalet de Llobregat, 08907 Barcelona, Spain; ^12^Department of Cardiology, Hospital Txagorritxu, Jose Achotegui s/n, Vitoria, 01009 Alava, Spain; ^13^Institute of Health Sciences (IUNICS), University of Balearic Islands and Hospital Son Espases, Carretera de Valldemossa 79, 07120 Palma de Mallorca, Spain; ^14^Department of Cardiology, Hospital Universitario Son Dureta, Andrea Doria 55, 07014 Palma de Mallorca, Spain; ^15^Department of Nutrition and Bromatology, School of Pharmacy, University of Barcelona, Avenida Joan XXIII s/n, 08028 Barcelona, Spain; ^16^Group of Nutrition and Lipid Metabolism, Instituto de la Grasa (CSIC), Avenida Padre García Tejero 4, 41012 Seville, Spain; ^17^CIBER Epidemiología y Salud Pública (CIBERESP), Instituto de Salud Carlos III, Melchor Fernández Almagro 3-5, 28029 Madrid, Spain; ^18^Lipid Clinic, Endocrinology and Nutrition Service, Hospital Clinic, IDIBAPS, Villarroel 170, 08036 Barcelona, Spain

## Abstract

*Objective*. To compare the one year effect of two dietary interventions with MeDiet on GL and GI in the PREDIMED trial. *Methods*. Participants were older subjects at high risk for cardiovascular disease. This analysis included 2866 nondiabetic subjects. Diet was assessed with a validated 137-item food frequency questionnaire (FFQ). The GI of each FFQ item was assigned by a 5-step methodology using the International Tables of GI and GL Values. Generalized linear models were fitted to assess the relationship between the intervention group and dietary GL and GI at one year of follow-up, using control group as reference. *Results*. Multivariate-adjusted models showed an inverse association between GL and MeDiet + extra virgin olive oil (EVOO) group: *β* = −8.52 (95% CI: −10.83 to −6.20) and MeDiet + Nuts group: *β* = −10.34 (95% CI: −12.69 to −8.00), when comparing with control group. Regarding GI, *β* = −0.93 (95% CI: −1.38 to −0.49) for MeDiet + EVOO, *β* = −1.06 (95% CI: −1.51 to −0.62) for MeDiet + Nuts when comparing with control group. *Conclusion*. Dietary intervention with MeDiet supplemented with EVOO or nuts lowers dietary GL and GI.

## 1. Introduction

The Mediterranean diet (MeDiet) is a unique plant-based dietary pattern, an expression of different food cultures of the Mediterranean region, and a group of practices, representations, knowledge, and skills that Mediterranean population have historically built and recreated in a sustainable interaction with nature [1–3].

Scientific research has established a beneficial role for the major components of the MeDiet, such as fatty acids, vitamins, minerals, fiber, and bioactive compounds in cardiovascular diseases (CVD) and other chronic degenerative conditions [4–7]. However, the possible beneficial role of carbohydrates has been little studied in comparison with existing literature regarding different types of fat [8]. Actually, there is epidemiological evidence linking consumption of whole grains and characteristics of the MeDiet, with decreased risk of CVD and type 2 diabetes mellitus (T2DM) [9].

It has been known for some time [10] that healthy and diabetic subjects show different postprandial glycemic responses to carbohydrates of different quality. Dietary glycemic index (GI) is an indicator of the quality of carbohydrates consumed in terms of glycemic response, while glycemic load (GL), the mathematical product of the GI of a food and its carbohydrate content, integrates the quantity and quality of carbohydrates consumed [11–13]. These two indices have been used in epidemiological studies to evaluate associations with chronic disease risk, but results have not been fully consistent. In a meta-analysis of 37 prospective studies [14], the effect of GL and GI on chronic diseases in general was modest, but more evident for T2DM, coronary heart disease, and gallbladder disease. A more recent meta-analysis of 8 prospective studies focusing on coronary heart disease showed that higher dietary GL and GI significantly increased risk among women and the unfavourable effects were more pronounced in overweight and obese subjects [15].

Many foods that are at the core of the MeDiet have a low GI, such as fruits, vegetables, legumes, nuts, and seeds, and this could play a role in the salutary effects of this dietary model, but only a few studies have reported the blood glucose response to carbohydrate-rich foods typically consumed in the Mediterranean area [16]. This prompted us to explore the effect of two dietary interventions with MeDiet on GL and GI in the PREDIMED study (Prevención con Dieta Mediterránea) [17] and evaluate their relationship with adherence to the MeDiet at one year of follow-up.

## 2. Methods

### 2.1. Study Design

This report is a longitudinal analysis within the frame of the PREDIMED study, a dietary intervention, parallel group, multicenter, single-blinded, randomized trial, designed to ascertain whether a MeDiet supplemented with olive oil or MeDiet supplemented with nuts prevents major cardiovascular events (cardiovascular death, myocardial infarction, and/or stroke) compared with a low-fat diet in a high risk population.

Participants were randomly assigned to one of the three diet groups by using a computer-generated random-number sequence. Subjects allocated in the MeDiet groups received individual and group dietary training at baseline and every three months during intervention. Additionally, participants received supplemental foods at no cost; extra virgin olive oil (1 L/week) was provided to the first group (MeDiet + EVOO) and 30 g/day of mixed nuts (15 g walnuts, 7.5 g hazelnuts, and 7.5 g almonds) to the second group (MeDiet + Nuts). The group sessions with the MeDiet groups were run by PREDIMED registered dietitians with up to 20 participants per session and separate sessions for each group. Each session consisted of informative talks and provision of written material with elaborate descriptions of typical MeDiet foods and seasonal shopping lists, meal plans, and cooking recipes. In the control group, dietary advice with the same periodicity and methods and a leaflet recommending the National Cholesterol Education Program Adult Treatment Panel III dietary guidelines were provided. Full PREDIMED protocol has been described elsewhere [17, 18]. The Research and Ethic Committee of the Hospital Clinic in Barcelona, Spain, accredited by the Department of Health and Human Services and regulated by the Federalwide Assurance for the Protection of Human Subjects of International (Non-US) Institutions no. 00000738 approved the study protocol. This trial has been registered in the London Current Controlled Trials ISRCTN 35739639. Written informed consent was provided by all participants.

### 2.2. Study Population

The original study sample included 7447 participants, aged 55 to 80 y, at high risk of CVD, with at least one of the following criteria: presence of T2DM, ≥3 cardiovascular risk factors (current smoking, hypertension, and high LDL cholesterol (≥160 mg/dL), low HDL cholesterol (≤40 mg/dL in men and ≤50 mg/dL in women), overweight or obesity (BMI ≥ 25 kg/m^2^), or family history of premature CVD. We assessed dietary GL and GI in nondiabetic subjects at baseline (3,833). Incident cases of diabetes at 1 year of follow-up were excluded (*n* = 61). Subjects with incomplete dietary data at baseline (*n* = 37) and at one year of follow-up (*n* = 773) were also excluded. Finally, we excluded subjects with values of total energy intake outside predefined limits at baseline (*n* = 63) and at one year of follow-up (*n* = 33). Overall, 2866 participants (1085 men and 1781 women) were analyzed in this study. We excluded participants with T2DM since this population usually has nutritional recommendations to follow a low GL/GI diet and could lead to a bias in our analysis.

### 2.3. Dietary Assessment

Information on dietary habits was collected by trained dietitians in face-to-face interviews using a 137-item food frequency questionnaire (FFQ) that was previously validated for a similar population [19]. In spite of the fact that FFQ was not designed to evaluate dietary GL and GI, intraclass correlation coefficients between two repeated FFQ were 0.85 for GL and 0.32 for GI. From the information collected in the FFQ, the daily intake of 131 food and beverage items was obtained in grams per day. Spanish food composition tables were used to estimate energy (kcal per day) and nutrient (grams per day) intake [20].

### 2.4. Assessment of the Adherence to the MeDiet

A 14-item dietary screener was used to assess adherence to the MeDiet at baseline and every three months [21, 22]. This tool was useful in evaluating the compliance with MeDiet, allowing personalized dietary advice to be provided to subjects allocated in MeDiet groups by adapting it to the participant's clinical condition, preferences, and beliefs [23]. This questionnaire consists of 14 dichotomous questions on food consumption frequency. Twelve questions set a cut-off point expressed in serving units per day or per week and indicate the typical serving size for each food. Each question was scored 0 or 1. One point was given for the use of olive oil as the principal source of fat for cooking, for preferring white meat over red meat, and for each of the following 12 food consumption patterns: (a) 4 or more tablespoons of olive oil per day (including that used in frying, salads, meals eaten away from home, etc.), (b) 2 or more servings of vegetables per day, (c) 3 or more pieces of fruit per day, (d) less than 1 serving of red meat or sausages per day, (e) less than 1 serving of butter, cream, or margarine per day, (f) less than 1 cup of sugar-sweetened beverages per day, (g) 7 or more servings of red wine per week, (h) 3 or more servings of pulses per week, (i) 3 or more servings of fish per week, (j) fewer than 2 commercial pastries per week, (k) 3 or more servings of nuts per week, and (l) 2 or more servings per week of “sofrito” which is a Mediterranean sauce made with onion, garlic, tomato, and spices sautéed in olive oil. If the condition was not met, 0 points were recorded for any particular item. The final score thus ranged from 0 to 14 points [21].

### 2.5. Estimation of Dietary GL and GI

We determined the amount of digestible carbohydrates in each meal using Spanish food composition tables [24]. Then, GI of each food present in the FFQ was assigned through a protocol described by Louie et al. [25], using data available in the International Tables of GI and GL Values 2002 [26] and the Sydney University GI research service [27]. Published values were extracted from studies in normal subjects, using 50 g of glucose as reference food and 2 hour testing periods [26]. The GI assignment for each food of the FFQ was performed as follows. Step 1: we determined whether there was a direct link to a food in a GI database with GI values obtained from studies conducted on healthy subjects. Of the 131 food items of the FFQ, the GI values of 60 foods were assigned. Step 2: a GI value of 0 was given to 59 food items due to their low carbohydrate content (<5 g of carbohydrates per 100 g). Step 3: we searched for a “closely related food item” in the databases used. The GI of 10 foods was specified according to values of related items from the database. Step 4: we determined whether the median GI value of the food subgroup was available. The median group in the food group of pastries and cakes was determined and assigned for 1 item. Step 5: finally, for the rest of the items that were not assigned with a GI value in the previous steps, we evaluated whether or not the item was a “top carbohydrate contributor.” If so, a GI value of 0, 50 or a GI value of an appropriate closely matched item as decided by the research nutritionists was assigned. Only one food item reached this stage and it was assigned with a GI value of 0 because it was not one of the main contributors of carbohydrates to the diet. After the GI assignment, we estimated dietary GL and GI by the following equations:

(1) $$\begin{array}{c} \text{Dietary}\;\;\text{GL}=\frac{\sum_{i=1}^{n}\left[{\text{GI}}_{i}\times {\text{CHO}}_{i}\right]}{100}\\ \text{Dietary}\;\;\text{GI}=\frac{\sum_{i=1}^{n}\left[{\text{GI}}_{i}\times {\text{CHO}}_{i}\right]}{\sum_{i=1}^{n}{\text{CHO}}_{i}}, \end{array}$$

where GI_*i*_ is the value of the food “*i*” obtained from the GI database; CHO_*i*_ is the amount of available carbohydrates from food “*i*” (g/g) multiplied by food intake (grams per day). And “*n*” is the number of foods consumed per day [13].

### 2.6. Other Measurements

Sociodemographic and lifestyle information was collected via specific questionnaires. BMI was estimated as weight (kg) divided by the square of height (m^2^). Leisure time physical activity was appraised using the validated Spanish version of the Minnesota Questionnaire [28, 29].

### 2.7. Statistical Analysis

We performed a descriptive analysis of baseline population characteristics according to intervention group. Qualitative and quantitative variables among groups were compared by using chi-square tests and analysis of variance (ANOVA), respectively. The intakes of carbohydrate, protein, fat, alcohol, and fiber and dietary values of GL and GI were adjusted by the residuals method proposed by Willett et al. [30].

In order to describe dietary GL and GI of our population at 1 year of follow-up, sample was distributed in quintiles of adherence to the MeDiet. Means were compared using ANOVA and *P*-trend was estimated using ANOVA-trend.

Generalized linear models were fitted to assess the relationship between the intervention groups and changes in dietary GL and GI at one year of follow-up, using the control group as reference. Multiple linear regression models were constructed to assess the relationship between adherence to MeDiet and GL and GI according to tertiles of adherence to MeDiet considering the lowest adherence as reference. The basic model was unadjusted. In two further models, the associations were adjusted for potential confounders including sex, age, physical activity (continuous), smoking (nonsmokers, smokers), total energy intake (continuous), and BMI (continuous). Analyses were performed using SPSS Software (version 18, 2009, SPSS Inc.) and a *P* value < 0.05 was considered statistically significant.

We explored the effect of this intervention at one year of follow-up because in this clinical trial, trained dietitians achieved a high adherence to the MeDiet in such period of time [23]. Participants allocated to both MeDiet groups increased their intake of low GI foods: virgin olive oil, nuts, vegetables, legumes, and fruits (*P* < 0.05 for all within- and between-group differences). Participants in all three groups decreased their intake of meat and pastries and cakes and sweets (*P* < 0.05 for all) [23].

## 3. Results

In Table 1 are presented the baseline characteristics of the population according to the study group. Subjects in the MeDiet groups were more likely to be younger and more physically active than those in the control group. In this study, subjects allocated in both MeDiet groups had slightly higher adherence to MeDiet and higher values of total energy intake than those in the control group. Carbohydrate consumption was higher in the control diet than both MeDiet groups' intakes. MeDiet groups consumed more polyunsaturated fat than the control group and MeDiet + Nuts group had higher alcohol intake than control diet. Dietary GL was lower in the MeDiet + Nuts group than in the control group. Dietary GI was similar in the three groups.

In this study, the mean GL was 118.0 (24.2) and average GI at baseline was 57.5 (4.7). Average GL in women and men was 118.5 (22.3) and 117.3 (27.0), respectively. Mean GI in women and men was 56.7 (4.6) and 58.8 (4.6), respectively.

Table 2 exhibits the regression coefficients and 95% CI for GL and GI changes according to the intervention groups at one year of follow-up. The multivariate-adjusted models showed a significant inverse association between GL and GI with MeDiet + EVOO (for GL *β* = −8.52; 95% CI: −10.83 to −6.20 and for GI *β* = −0.93; 95% CI: −1.38 to −0.49) and a more pronounced effect in the MeDiet + Nuts group (for GL *β* = −10.34; 95% CI: −12.69 to −8.00 and for GI *β* = −1.06; 95% CI: −1.51 to −0.62), when compared to control diet.

**Figure 1:**
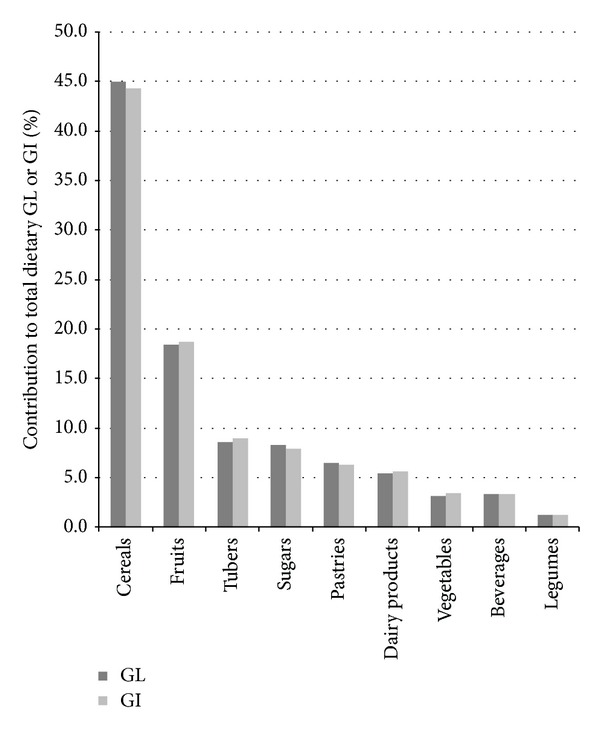
Relative contribution of food groups (%) to total dietary GL and GI in nondiabetic subjects in the PREDIMED study at one year of follow-up.

Figure 1 illustrates the relative contribution of food groups to dietary GL and GI at one year of follow-up. The group of cereals and cereal products was the main contributor, providing 45.0% to the total dietary GL and 44.3% to total dietary GI. Within this cereals group, the contribution of GL for white and whole grain bread was 28.0% and 6.9%, respectively. Regarding GI, white bread supplied 26.9% of total dietary GI, followed by whole grain bread with 7.2%. The fruit group supplied around 18.0% of total dietary GL and GI. Tubers, particularly potatoes, contributed 8.6% and 9.0% to total GL and GI, respectively. In this population, 8.3% and 7.9% of total dietary GL and GI were provided by the group of simple sugars. The contribution of legumes to total dietary GL and GI was minor, as they supplied only 1.2% of GL and 1.3% of GI.

**Figure 2:**
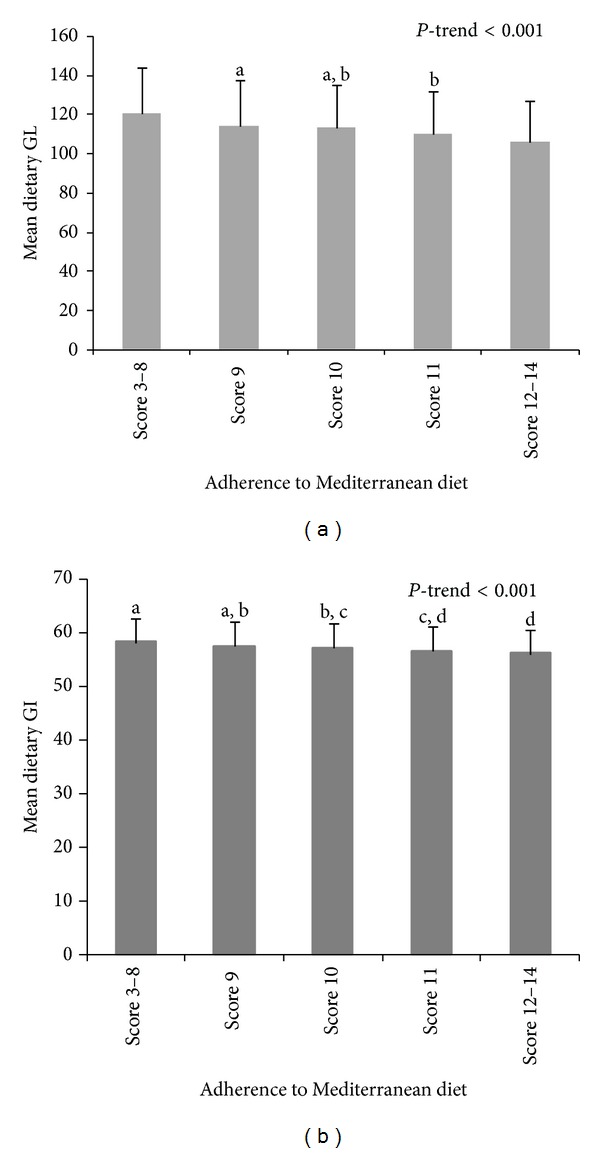
Mean dietary GL and GI quintiles according to the adherence to Mediterranean diet in the PREDIMED study at one year of follow-up. Means with superscripts without a common letter differ, *P* < 0.05.

In order to describe in detail dietary GL and GI of the population, we divided the sample into five categories of adherence to MeDiet. The mean (SD) GL of the highest adherence group was significantly lower than those of the rest of categories (106.1 (20.5); *P* for trend < 0.001) (Figure 2(a)). Similarly, the mean (SD) GI for the highest category was significantly lower than that of the first category (56.0 (4.4) and 58.1 (5.0), resp.) (Figure 2(b)). We found a significant linear trend for dietary GI across categories of adherence to MeDiet (*P* for trend < 0.001).

Table 3 shows the regression coefficients with 95% confidence intervals (CI) of GL and GI values by categories of adherence to the MeDiet. The unadjusted linear regression model showed significant negative associations between dietary GL and the second and third categories of adherence to the MeDiet compared to the lowest (reference) category. The results remained significant after adjustment for possible confounders. Similar results were found regarding dietary GI.

## 4. Discussion

In a large sample of older nondiabetic subjects at high risk of CVD participating in the PREDIMED study, we found that an intervention with MeDiet supplemented either with EVOO or nuts lowers the GL and GI of the diet. We observed that changes in dietary GL after one year of intervention were more pronounced than dietary GI changes, because GL contemplates both the quality and amount of carbohydrates consumed. Although there have been several studies describing GL and GI in different countries and population groups [12, 13, 31], to our knowledge, this relationship had not been assessed before.

In this study, an inverse association was found between GL/GI and MeDiet + EVOO when compared with the control group. Similar results were found for MeDiet + Nuts. This could be explained due to intervention in both MeDiet groups, which included dietary advice to increase consumption of vegetables, fruits, legumes, fish, olive oil, and nuts (in the MeDiet + Nuts groups), and these foods frequently consumed in the MeDiet have a low GI. On the other hand, patients were advised to reduce their sweetened and/or carbonated beverages intake and commercial sweets or pastries intake, therefore, reducing the servings of foods with high GI values. Furthermore, studies have demonstrated that common foods such as spaghetti or potato dumplings have a low GI in spite of their low fiber content [16]. However, in our study population, white bread and whole grain bread supplied a considerable proportion of the total dietary GL and GI (around 35%). Previous studies have shown that bread consumption in a MeDiet pattern, especially whole grain bread, has a beneficial effect on adiposity [32], while increased consumption of white bread has the opposite effect [33].

In our study, the estimated mean dietary GL at baseline was 118 and the mean dietary GI was 58. These values differ from those reported in other studies. Thus, the dietary GL and GI in a Spanish population sample were lower compared with our study, probably because of a wider age range [34]. In the EPIC study, the mean GL was also higher, which could be explained by a higher intake of carbohydrate (222 (31) g/day) in the population studied [35].

The MeDiet is a plant-based dietary pattern. Plant foods are the main source of nutrients such as slow-release carbohydrate and fiber, vegetable protein, beneficial minerals, antioxidant vitamins, and polyphenols that contribute to an optimal nutrition, satiety, and maintenance of a balanced diet. The group of cereals is found in the main meals, preferably consumed as whole grains, emphasizing the importance of a high fiber content in the diet [1, 3, 36]. In our study, greater fiber intake in MeDiet groups may be related to lower dietary GL and GI. A recent meta-analysis reported that many interventions directed at lowering the GI of a diet also resulted in increased intakes of fiber, usually causing a decreased GL and that GL acts independently from fiber on fasting blood glucose [37].

The relevance of assessing the effect on GL and GI relies on their relationship with chronic disease, assessed in several studies. For instance, high dietary GL and GI were associated with an increased risk of coronary heart disease events in women [14, 38]. Also, a recent meta-analysis showed a significant association between GL and GI and the risk of colorectal and endometrial cancer, while the relationship with other types of cancer was inconsistent [39]. Moreover, it has been demonstrated that diets with lower values of GL and GI reduce T2DM risk [40] with a protective effect similar to that of whole grain and high fiber intakes [14]. Recently, the combined effect of dietary GL and MeDiet on the risk of incident T2DM has been evaluated in Greek population. Adherence to the traditional MeDiet was assessed through the MeDiet score (MDS). The authors found a positive but weak correlation between MDS and GL (*r* spearman = 0.28). However, subjects with a high MDS and a low GL tended to have an 18% lower risk of diabetes when compared with participants with a diet characterized by a high GL and a low MDS [41].

Furthermore, various similar clinical trials have evaluated the effect of a modified Mediterranean-style low GL diet on prevalence of metabolic syndrome (MetS) components in women. The first study showed a significant reduction of MetS components [42]. The second study demonstrated beneficial changes in waist circumference, plasma triglycerides, LDL cholesterol, and systolic blood pressure in female participants [43]. Significant decreases were also found in plasma insulin, TNF-*α* levels, and HMG-CoA reductase expression. The latter finding indicates decreased cholesterol biosynthesis, although the trigger is unclear [43]. In the third study, a significant increase in plasma lutein and *β*-carotene was found after 12 weeks. In addition to LDL cholesterol reduction, decreases were observed in some atherogenic subfractions with different particle diameters: large very low-density lipoprotein (VLDL, >60 nm), small LDL (18–19.8 nm), and medium high-density lipoprotein (8.2–8.8 nm). [44]. In addition, another investigation showed that Apolipoprotein B was reduced in women with a low GL and Mediterranean-type diet [45]. Also, the MeDiet with a low GL seems to have beneficial effects on weight, waist circumference, and systolic blood pressure [46].

Moreover, a MeDiet supplemented with nuts during 1 year was associated with a 14% reduction in prevalence of MetS [47]. A cross-sectional study in the Canary Islands showed that fruit intake had a protective effect on the triglyceride criteria of MetS and that cereal intake protected from insulin resistance [48]. Recently, it was found that MeDiet supplemented with extra virgin olive oil or nuts can reduce by 30% the incidence of major cardiovascular events when comparing to a low-fat diet [18].

The mechanisms of action by which low GL/GI diets possibly decrease chronic disease risk could be related to postprandial glucose and its related endocrine responses. After consumption of a high GI food, a dramatic increase in blood glucose occurs; this is followed by a large insulin response and inhibition of glucagon release. On the contrary, intake of low GI foods results in an attenuated glucose response due to delayed intestinal glucose absorption. Therefore, the resulting hormone responses and their effects are more stable, reducing postprandial hyperglycemia and hyperinsulinemia and attenuating late postprandial rebounds in circulating nonesterified fatty acids. These effects of a low GI diet could attenuate oxidative stress, which is associated with inflammation, and other risk factors [49]. Although low GL and GI have been associated with a reduced risk of chronic diseases, there is still insufficient evidence to include them in dietary recommendations to the general population [50].

The main strengths of this study are its large sample size, use of a comprehensive and validated FFQ with assignment of GI values through an established protocol, use of a validated MeDiet screener, and adjustment for all possible confounders in multivariate analyses.

Our study also has limitations. As it was conducted in older individuals at high CVD risk, the results cannot be easily extrapolated to other populations. Another limitation is that values of GI for Spanish food are scarce; we assigned them using general GI tables obtained mainly from Australian and US studies; estimations could be misrepresented because the properties of foods with the same name may fluctuate between countries.

## 5. Conclusions

In summary, our findings suggest that MeDiet supplemented with EVOO or nuts lowers the GL and GI of the diet. At any rate, GL and GI of the MeDiet could explain other mechanisms for protection against CVD apart from the MUFA/SFA ratio [51] and the antioxidant capacity [5] involved in this beneficial dietary pattern. More researches are needed to clarify the role of these indexes in future dietary recommendations.

## Figures and Tables

**Table 1:** Baseline characteristics of participants according to the intervention group in the PREDIMED study^a^.

	Control diet	Mediterranean diet with EVOO	Mediterranean diet with nuts	*P*
	*n* = 822	*n* = 1051	*n* = 993	
Sex (%)				
Female	64.1	62.4	60.2	0.229
Age (years)	67.2 (6.1)	66.5 (6.0)	66.5 (5.9)	0.020
BMI (kg/m^2^)	30.1 (3.7)	30.0 (3.6)	29.7 (3.6)	0.090
Physical activity (METS/d)	209.5 (218.8)	233.4 (216.4)	243.7 (231.6)	0.004
Smoking (%)				
Never	61.6	62.5	63.5	0.930
Past	23.2	22.5	21.6	
Current	15.2	15.0	14.9	
Score of adherence to MeDiet	8.5 (1.9)	8.9 (1.9)	8.9 (1.9)	<0.001
Energy intake (kcal/d)	2201 (527)	2317 (532)	2312 (514)	<0.001
Carbohydrates (g/d)^b^	247.4 (40.1)	245.5 (41.1)	241.0 (39.5)	0.002
Protein intake (g/d)^b^	90.3 (12.7)	91.5 (13.9)	91.3 (13.4)	0.160
Total fat intake (g/d)^b^	96.6 (16.7)	97.1 (17.1)	98.1 (15.9)	0.160
Monounsaturated fat	48.0 (11.3)	48.7 (10.9)	48.8 (10.3)	0.220
Polyunsaturated fat	15.5 (4.8)	15.2 (4.8)	16.0 (5.2)	0.002
Saturated fat	24.5 (5.8)	24.4 (5.9)	24.7 (5.5)	0.530
Alcohol intake (g/d)^b^	8.0 (13.5)	9.0 (14.5)	10.3 (16.0)	0.004
Fiber intake (g/d)^b^	25.4 (7.8)	25.8 (8.2)	25.7 (7.5)	0.620
Dietary GL^b^	119.6 (23.8)	118.9 (25.1)	115.8 (23.3)	0.001
Dietary GI^b^	57.7 (4.8)	57.6 (4.8)	57.3 (4.5)	0.160

^a^Continuous variables are showed as means (SD) and categorical variables are expressed as percentages.

^b^ Energy adjusted by residuals method.

**Table 2:** Regression coefficients and 95% CI for GL and GI changes according to the intervention group at one year of follow-up (*n* = 2866).

	Intervention group		
	Control diet	Mediterranean diet with EVOO	Mediterranean diet with Nuts
Change in dietary GL^a^			
Model 1	0 (Ref.)	−8.62 (−11.00 to −6.28)	−10.36 (−12.73 to −7.99)
Model 2^b^	0 (Ref.)	−8.66 (−11.00 to −6.32)	−10.44 (−12.82 to −8.07)
Model 3^c^	0 (Ref.)	−8.52 (−10.83 to −6.20)	−10.34 (−12.69 to −8.00)
Change in dietary GI^a^			
Model 1	0 (Ref.)	−0.86 (−1.30 to −0.42)	−1.00 (−1.45 to −0.56)
Model 2^b^	0 (Ref.)	−0.85 (−1.30 to −0.41)	−0.99 (−1.44 to −0.54)
Model 3^c^	0 (Ref.)	−0.93 (−1.38 to −0.49)	−1.06 (−1.51 to −0.62)

^a^Changes were estimated from baseline to one year of follow-up.

^b^Adjusted for age (years) and gender.

^c^Adjusted for age (years), gender, total energy intake (g/d), and energy adjusted fibre intake (g/d).

**Table 3:** Regression coefficients and 95% CI for GL and GI according to categories of adherence to MeDiet pattern at one year of follow-up (*n* = 2866).

Variables	Adherence to Mediterranean diet pattern			*P* for trend
	Low adherence	Moderate adherence	High adherence	
	(score 3–9)	(score 10-11)	(score 12–14)	
Dietary GL				
Model 1^a^	0 (Ref.)	−6.25 (−8.16 to −4.35)	−11.75 (−13.85 to −9.65)	<0.001
Model 2^b^	0 (Ref.)	−6.33 (−8.23 to −4.43)	−11.84 (−13.94 to −9.75)	<0.001
Model 3^c^	0 (Ref.)	−6.82 (−8.75 to −4.88)	−13.00 (−15.18 to −10.83)	<0.001
Dietary GI				
Model 1^a^	0 (Ref.)	−0.97 (−1.37 to −0.57)	−1.80 (−2.24 to −1.36)	<0.001
Model 2^b^	0 (Ref.)	−1.05 (−1.44 to −0.66)	−2.00 (−2.43 to −1.56)	<0.001
Model 3^c^	0 (Ref.)	−0.93 (−1.33 to −0.54)	−1.87 (−2.32 to −1.43)	<0.001

^a^Dietary GL and GI were energy adjusted by residuals method.

^b^Adjusted by age (years) and gender.

^c^Adjusted by age (years), gender, smoking (nonsmokers, smokers), total energy intake (g/d), physical activity (continuous), and BMI (continuous).
